# Metal artifacts with dental implants: Evaluation using a dedicated CT/MR oral phantom with registration of the CT and MR images

**DOI:** 10.1038/s41598-018-36227-0

**Published:** 2019-01-24

**Authors:** Min-Young Lee, Kyu-Ho Song, Jeong-Woo Lee, Bo-Young Choe, Tae Suk Suh

**Affiliations:** 1 Department of Biomedical Engineering, College of Medicine, The Catholic University of Korea, Seoul, 06591 Republic of Korea; 2 Department of Biomedicine & Health Sciences, The Catholic University of Korea, Seoul, 06591 Republic of Korea; 3 Research Institute of Biomedical Engineering, College of Medicine, The Catholic University of Korea, Seoul, 06591 Republic of Korea; 4 Department of Radiation Oncology, Konkuk University Medical Center, Seoul, 05030 Republic of Korea

## Abstract

The aims of this study were to develop a computed tomography/magnetic resonance (CT/MR) oral phantom with insertable dental implants and to register CT/MR images to generate artifact-free MR images for patients undergoing teeth restorations. All measurements were done using a human MR scanner with spin echo (SE) and gradient echo (GRE) sequences image scan together with CT image. The metal regions and normal teeth parts are extracted with a suitable threshold from an initial image reconstructed with artifact from the CT images. Corrected metal projection regions of MR images and CT images are fused to produce artifact-free MR image that include dental restorations. After CT/MR registration, artifact size presented differences on the x- (SE, 12.0 mm; GRE, 18.0 mm) and y- (SE, 24.0 mm; GRE, 36.6 mm). When comparing the dental restoration with normal teeth, the structural similarity index metric (SSIM) of GRE 50 was lower than for the GRE 8 sequence and the SSIM of SE 145 shown higher than for the SE 490 sequence. The dedicated phantom provides a useful tool in head and neck research for multi-modality images. Therefore, CT/MR image-based approach for ground truth and registration offers visualization in diagnostic system and radiation treatment planning system.

## Introduction

Computed tomography (CT) is the standard imaging modality used for patients with head and neck cancers because its high spatial resolution provides all the necessary information in a single series of images^1^. However, the streaks and dark bands caused by dental restorations do not allow visualization of the anatomical structures, and therefore, reduce the diagnostic value of the CT images^2^. In comparison, magnetic resonance (MR) imaging is helpful in such cases, where the distinction between tissue masses and the surrounding soft tissue structures is apparent, without using ionizing radiation. However, metal artifacts are also a significant feature of MR images. The magnetic susceptibility of dental amalgams causes image degradation, with magnetic field distortion and signal loss of the adjacent tissue, due to the strong magnetic field and radiofrequency (RF) used^3^. Further, due to the high magnetic susceptibility (>300 ppm; ferromagnetic materials) within the magnetic field, signal can accumulate in the region of interest (known as pile-up artifact), resulting in abnormally high signal^4^. In the human maxillofacial region, various metallic materials may increase image distortion, in terms of shape and orientation, which occurs due to the inhomogeneity in the static magnetic field caused by the metals, resulting in dephasing and signal loss^4,5^. Nevertheless, recently, the number of patients who have been treated with fixed orthodontic appliances, and referred for MR imaging, has increased^6^.

To reduce the metal artifacts with high magnetic susceptibility, these effects can be compensated by using one of the following: (a) spin echo (SE) sequence with modified parameters (i.e., echo time [TE], matrix, bandwidth, slice thickness, and echo train); (b) gradient echo sequence (GRE) with a very short TE^7,8^; (c) optimized metal artifact-free sequence^3,9^ with a specific intensity and size of susceptibility artifact (SA). Various methods have been developed for metal artifact-free in the last three decades, examining the effects of metal objects in different imaging modalities^10^. There are a few studies on methods of metal artifact-free, and the resulting error reduction, including signal loss and pile-up, which make it possible to substitute or estimate the true value of an object. However, errors made during and after image reconstruction prevent the true value of the objects from being estimated^11^.

Image registration allows researchers to overlay images from multiple imaging modalities, providing a more comprehensive representation of the anatomical and biological features, and overcoming the weakness of one specific modality. Multimodal imaging not only accelerates the radiotherapy planning process by identifying the disease^12^, including cancer delineation, but also offers excellent soft-tissue discrimination that improves the diagnosis of diseases, including head and neck cancers^13,14^, and infections^15^. The aims of this study were: (a) to develop a CT/MR oral phantom consisting of containers with insertable dental implants; (b) to provide guidance for the selection of sequences for acquiring images with fewer artifacts; and (c) to register CT/MR images to generate artifact-free MR images for patients undergoing teeth restorations. Based on phantom studies, we hypothesized that CT/MR oral phantom with two hemispheric containers would be registered CT/MR images to generate metal artifact-free MR images with the teeth and implants included to detect teeth from MR image.

## Results

### Evaluation of the quality for MR images with metal artifacts

**Figure 1:**
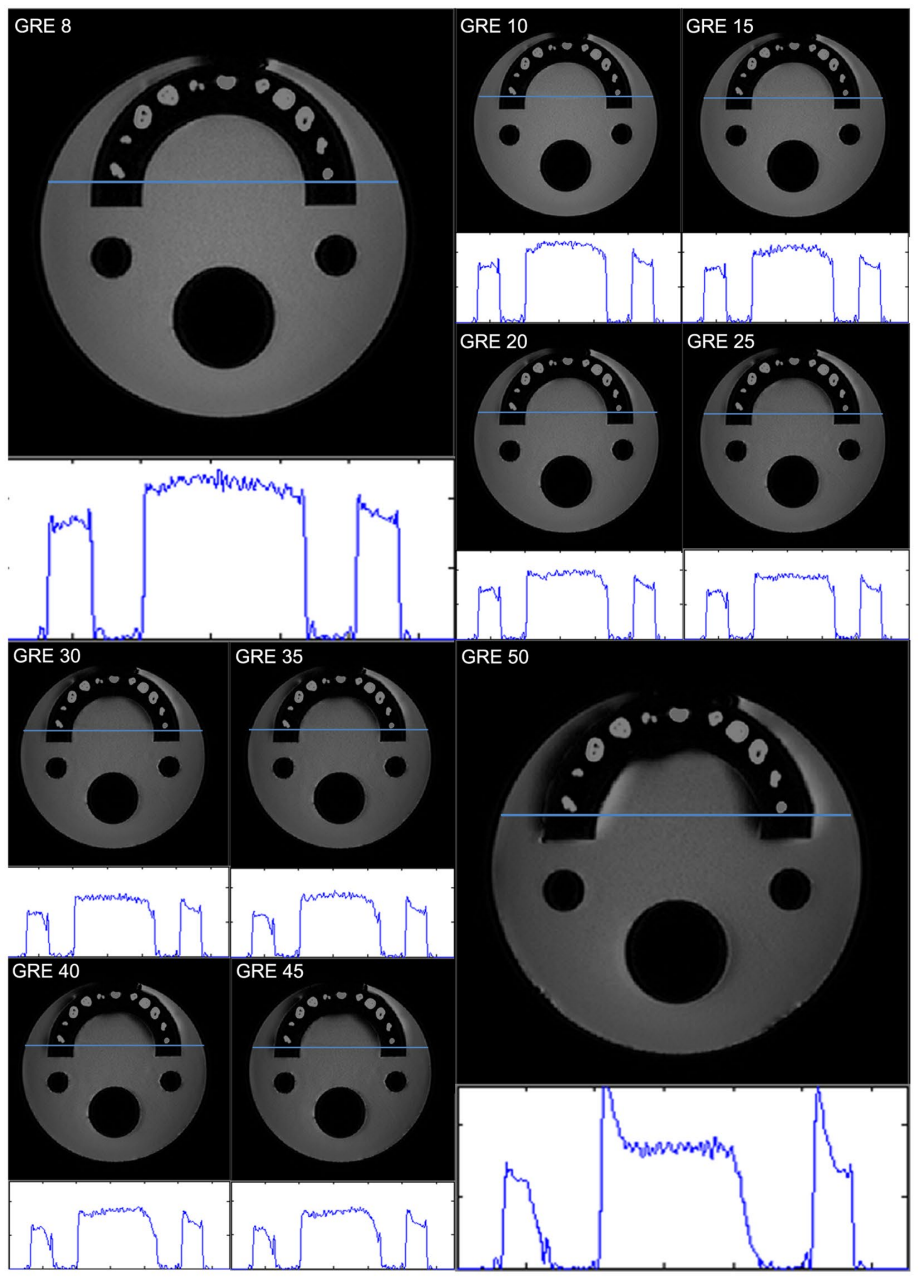
The signal intensity distribution on the line that drawn on the CT/MR registered image. The teeth are segmented from the top middle dental restoration CT image and registered on MR images with difference pulse sequences (3.0 T). Figures acquired comparison of signal intensity with different GRE sequences with same segmentation region registered.

**Figure 2:**
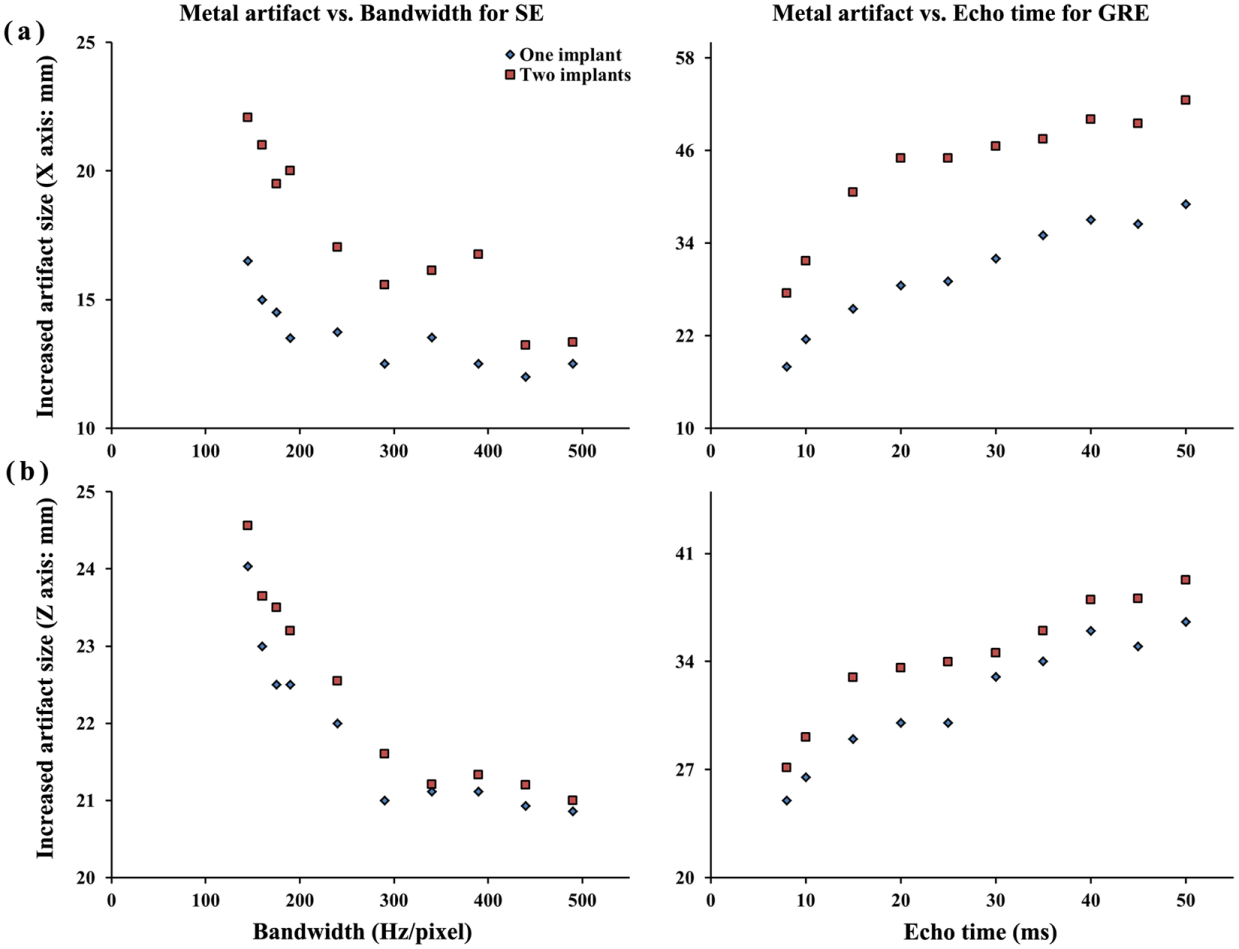
Increased artifact size in (**a**) x-axis and (**b**) z-axis dependence on bandwidth (SE, 140–490 Hz/px) and echo time (GRE, 8–50 ms).

The typical appearance of the metal artifacts during MR imaging is shown in Fig. 1. The phantom was used to assess the magnitude and spatial dependence of MR geometrical distortion in various sequences and CT images with artifacts. The results for the evaluated MR images, with one implant and two implants, are shown in Fig. 2. Each slice image from the center of the hemispherical phantom was used for evaluation and analysis of reproducibility using identical equipment (Magnetic Resonance Research, Korea Basic Science Institute, Korea). After stabilizing the dental restorations and teeth at various positions on the dental arch, the CT scans were obtained. The maximum artifact size differences (one implant) on the x- (SE, 12.0–16.5 mm; GRE, 18.0–39.0 mm) and z- (SE, 20.9–24.0 mm; GRE, 25.0–36.6 mm) axes were 39.0 mm and 36.6 mm, respectively. The increase in number of artifacts on the x- and y- axes was listed by the dependence on location of dental restoration and image sequences. However, as the artifacts are not spherically symmetric, the volumetric measurements of the artifact were estimated by drawing the contour based on the intensity gradient of the edge of the lesion automatically. The automated contour extraction contributes to high-quality segmentation. This method relies on the gradient information and the performance is completely dependent on the location of the initial contour, which must be as close to the region of interest^16,17^. For the SE sequences, the slices which contained maximum artifacts were used to determine the size of the artifact volumes with minimum of 4.9 ml, maximum of 458.34 ml, mean of 214.43 ml, median of 269.61 ml, and standard deviation of 118.2 ml. For GRE sequence, the slices which contained maximum artifacts were used to determine the sizes of the artifact volumes with minimum of 107.9 ml, maximum of 545.62 ml, mean of 137.34 ml, median of 81.84 ml, and standard deviation of 143.9 ml.

The physical density and effective atomic number can both be used for comparing phantom materials. The phantom used in this study accurately simulates the physical density of various human tissues. The patients’ mean Hounsfield units (HU) value for the dental restoration, and the HU value of the phantom were 2,976 HU and 3,095 HU, and that for the cervical vertebrae and the phantom were 998 HU and 1,013 HU, respectively. The mean HU error between the patients and the phantom was 2.95%.

### Image quality upon segmentation and registration

**Figure 3:**
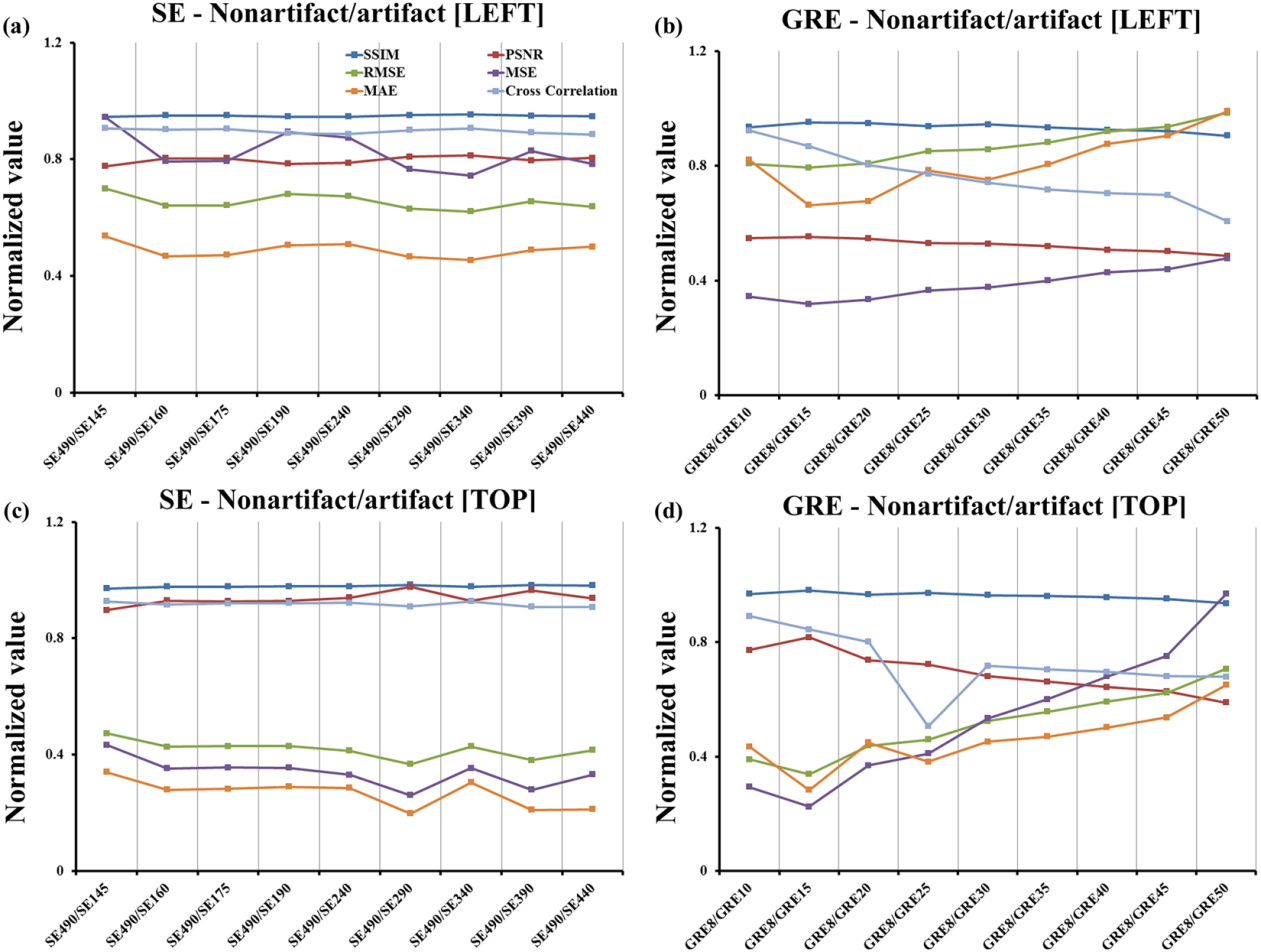
The comparison of the artifact-free CT/MR image with one dental restoration on the left side shown (**a**) with SE sequence and (**b**) with GRE sequence. The comparison of artifact-free CT/MR image with two dental restorations on top middle regions with (**c**) SE and (**d**) GRE sequences.

**Figure 4:**
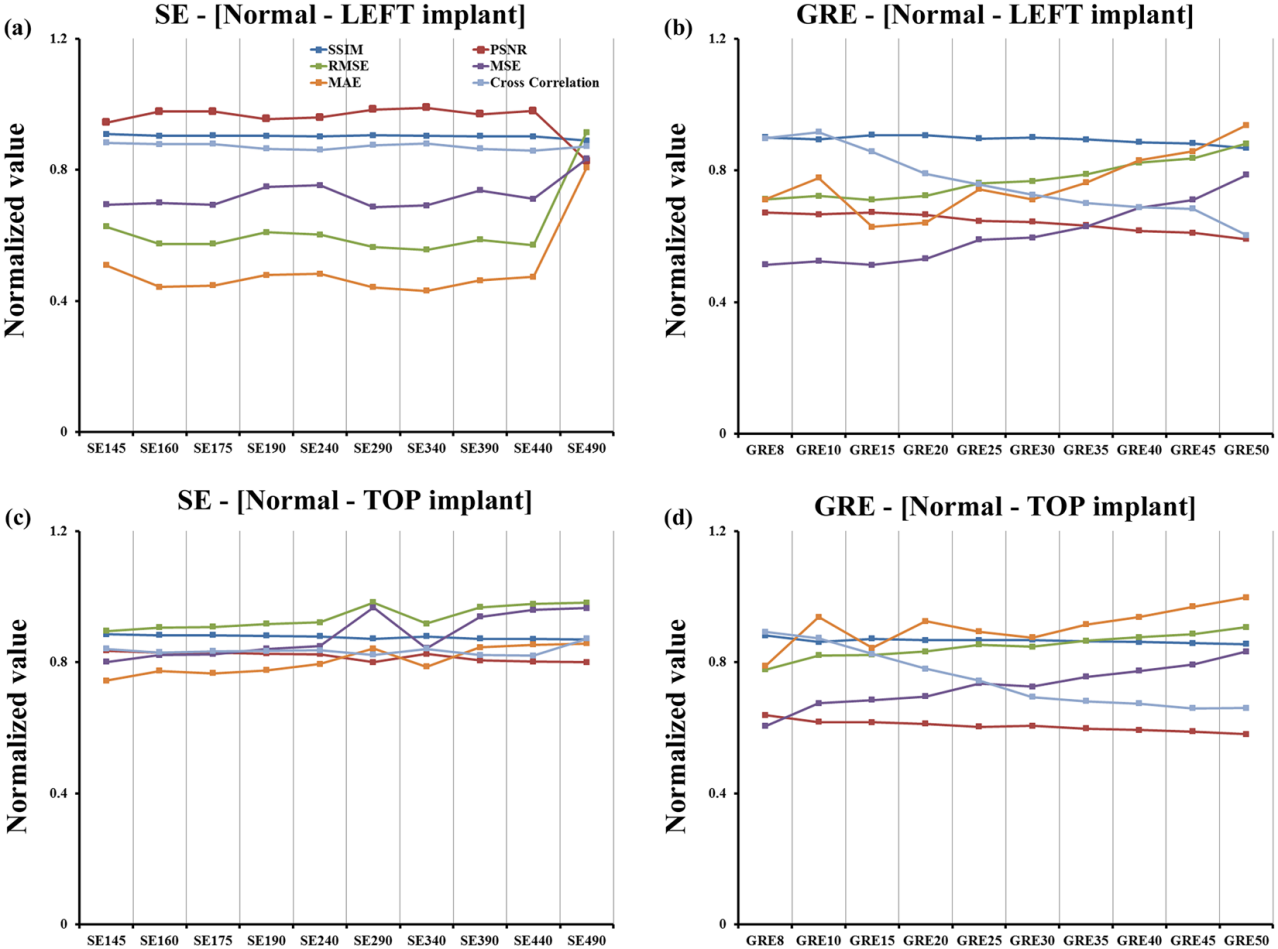
The comparison of the normal teeth (no implant) CT/MR image with one dental restoration on the left side shown with (**a**) SE and (**b**) GRE sequences. The comparison of normal CT/MR image with two dental restorations on top middle regions with (**c**) SE and (**d**) GRE sequences.

In Figs 3a,b and 4a,b, for GRE 50 (the GRE sequence that has an artifact), the mean-squared error (MSE) was 34.63% greater than that for the GRE 8 sequence. In GRE 50, the root mean square error (RMSE) was 19.16% higher than that for the GRE 8 sequence. The peak signal-to-noise ratio (PSNR) for GRE 8, which had a left dental restoration, was 11.97% higher than that of the PSNR for the GRE 50 image. A cross-correlation demonstrated that GRE 8 had a higher correlation coefficient value than that of GRE 50. The mean absolute error (MAE) of GRE 50 was 24.08% lower than that of GRE8. The similarity index metric (SSIM) is more accurate and consistent than MSE and PSNR. When comparing left dental restoration with normal teeth, the SSIM of GRE50 was 3.65% lower than for the GRE 8 sequence. For SE 145, which had an artifact, the MSE was 106% less than that of the MSE for SE 490. In SE 145, the RMSE was 31.47% higher than that for the SE 490 sequence. The PSNR of SE 440, which had a left dental restoration, was 9.26% lower than that of the PSNR of the SE 145 image. For cross-correlation, the SE 490 had a lower correlation coefficient value than the SE 145. Lastly, the MAE value for SE 145 was 19.51% higher than that for the SE 490 sequence. When comparing left dental restoration with normal teeth, the SSIM of SE 145 was 2.15% higher than for the SE 490 sequence.

In Figs 3c,d and 4c,d, for GRE 50, the MSE was 27.32% greater than the MSE for the GRE 8 sequence. The RMSE of GRE 50 was 14.36% higher than the RMSE for GRE 8. In the GRE 8 sequence, which had two top dental restorations, the PSNR was 9.17% higher than that of the GRE 50 image. Cross-correlation analysis showed that GRE 8 had a higher correlation coefficient value than GRE 50. Lastly, the MAE of GRE 50 was 21% lower than that of GRE 8. When comparing top dental restorations with normal teeth, the SSIM of GRE 50 was 3.00% lower than for the GRE 8 sequence. In SE 145, which had an artifact, the MSE was 17.01% less than that of SE 490, and the RMSE of SE 145 was 8.80% lower than that of SE490. Significant improvements were observed with bandwidth higher than 145 Hz/pixel. The PSNR for SE 490, which had a left dental restoration, was 4.17% lower than the PSNR for the SE145 image. A cross-correlation analysis showed that SE490 had a lower correlation coefficient value than that of SE 145. The MAE value for SE 145 was 18.48% higher than that for the SE 490 sequence. When comparing left dental restoration with normal teeth, the SSIM of SE 145 was 1.68% higher than for the SE 490 sequence.

## Discussion

In this study, we identified various parameter-dependent changes for the metallic artifacts-free by using a custom-made CT/MR oral phantom. We also attempted to register the CT/MR images to generate artifact-free MR images in the presence of dental restorations. The phantom allowed realistic visualization of the teeth and dental restorations, so that the ground true values were obtained from the images.

In radiology, specialized CT and MR phantoms containing organ-mimicking devices have been developed for the correction of geometric distortion^18^, including quality-control phantoms for dental cone-beam CT, multi-modality phantoms^19^, MRI-magnetic resonance spectroscopy (MRS) phantoms for quantitative evaluation^20^, and functional MRI phantoms^21^. We developed an acrylic phantom specifically designed to investigate the two main effects of metallic artifacts, signal loss and signal pile-up, in CT and MR imaging, and the field of view (FOV) was maintained in both modalities. There was no need to change the copper sulfate solution between the different scans (different position of dental restorations, sequence, and scan numbers). The phantom allowed realistic visualization of the teeth and dental restorations, so that the true values were obtained from the images. The teeth were set in thermoplastic resin to allow insertion of teeth and restorations in the desired positions. Thus, we designed and developed a spherical phantom with insertable dental restorations that helped us evaluate the image quality at high field strength. However, as the phantom in circular shape, there was the small amounts of air formed inside the acrylic phantom which was inevitable.

Widely used for high-resolution scans, CT is a low-noise imaging modality that shows the typical appearance of jaw anatomy, and dental diseases of the jaw. Dental CT has become an established method for imaging jaw anatomy, prior to the placement of dental restorations. It offers the advantage of accurate visualization of the dental scale, and therefore, allows exact measurement to determine the correct size of dental restorations. Additionally, CT has been used for determining palatal bone volume and the potential site for the placement of dental restorations^22^. Compared to CT, which usually helps define the contours of lesions, MR provides much greater soft-tissue definition and contrast. Although MR image quality at high field strength has improved, as for all modalities (i.e., single photon emission computed tomography, positron emission tomography, CT, and ultrasound), artifacts should be prevented to minimize diagnostic errors.

Further, MR imaging has been used in the diagnosis of temporomandibular joint disorders and soft tissue pathologies, and in the placement of dental restorations. It demonstrates great precision in the measurement of bone composition and anatomy, and the volume of teeth or dental restorations. It also demonstrates great spatial resolution for investigating the functional aspects of temporomandibular joint dysfunction and its symptoms. It is important to obtain predictable results by clinical dental assessment. However, a dental restoration causes a magnetic field inhomogeneity, which in turn causes a local signal void, and is often accompanied by an area of high signal intensity, as well as a distortion of the image. The dental restorations must be suitable for MR imaging, and must lead to minimal susceptibility artifacts that may affect diagnostic information. In addition, the effects of dental restorations depend on shape, composition, and orientation in a high field strength MR system.

Metallic implants placed within the magnetic field lead to considerable distortion and generate spatial encoding errors in head and neck MR images^23^. The magnetic susceptibility of a metallic implant is described by how magnetized a material is within a magnetic field^24^. In addition, signal pile-up is usually observed as a high intensity signal of the curve adjacent to the blank area with the contour of the metallic implants. In the present study, the magnetic field inhomogeneity interfered with the linear magnetic field gradients, and led to spatial shifts of tissue signal near the dental restoration, which changed signal intensity. The image pixel shift was proportional to the susceptibility difference between objects, and was inversely proportional to the gradient of field strength. This suggests the importance of choosing pulse sequences and parameters that reduce artifacts due to metallic SA. In this study, an increased bandwidth resulted in a decreased signal to noise ratio (SNR). The SNR is inversely proportional to the square root of the bandwidth^4,23,24^. Further, since GRE sequence is very sensitive to field heterogeneity, the GRE image with a decreasing TE also had an increased SNR, due to T2^*^ relaxation. In the present study, an optimal protocol with minimal metallic SA was necessary.

The spatial shift of the signal from the area near the dental restoration is generally not the same for all pixels. Image segmentation is the process by which an image is divided into regions, or segments, based on various criteria. Various segmentation algorithms for medical images have been implemented in recent studies, including k-means, fuzzy c-means (FCM), Otsu’s threshold, and watershed. The main advantage of FCM is that it allows gradual allocation of data points to clusters measured as degrees, which implies that they may belong to more than one cluster. The FCM algorithm provides greater accuracy and efficiency of image threshold segmentation than other methods, needing fewer iterations before converging on an optimal solution^25,26^. The multi-level threshold technique enables segmentation of an image at many scales, and increases the chance of attaining better results. As there is a big difference according to the number of implants and implant localization, there would a difference in suppressing artifacts if implants were placed bilaterally or dentition restored with full mouth. This can be incorporated into future research with fabricated of a phantom with multiple implants. Also, we plan to extend our methods to enable treatment localization and to deliver a more accurate dose calculation.

Measurement of image similarity is important for image processing applications. Recently, many image quality assessment techniques have been studied^27–31^. There are two classes of image distortion assessment approaches, objective fidelity criteria and subjective fidelity criteria methods. Objective image quality metrics can be used to compare an image with a distorted image. The objective fidelity criteria are based on mathematically defined assessment approaches, including MSE, PSNR, MAE, and RMSE. The subjective fidelity criteria consider human visual system (HVS) characteristics, which contain SSIM, universal quality index (UQI), feature similarity, and gradient magnitude similarity (GMS). In this study, SSIM, which is based on HVS, was used to measure the image quality because UQI fails to correlate all subjective assessments resulting in instability. Further, GMS evaluates horizontal and vertical gradients of the reference and distorted images, but the gradient is sensitive to noise. We decided to use the developed phantom in 7 T MRI to find the differences of the occurrence of metal artifacts and utilize those images in radiation treatment planning system for future study.

In conclusion, a dedicated oral phantom is a unique and useful tool for studying head and neck regions, since it offers reference data for metal susceptibility artifact-free, and registration methods for multi-modal medical images. Additionally, the phantom may provide dental image guidance for patients with oral disease, in order to diagnose and treat the functional aspects of disc position, degree of disc displacement, disc deformity, joint effusion, and osteoarthritis. For patients with oral disease treated with a fixed orthodontic appliance, a contrast-enhanced dental MR image offers a means of visualizing the different anatomical structures in a diagnostic system.

## Methods

### Dedicated CT/MR oral phantom fabrication

**Figure 5:**
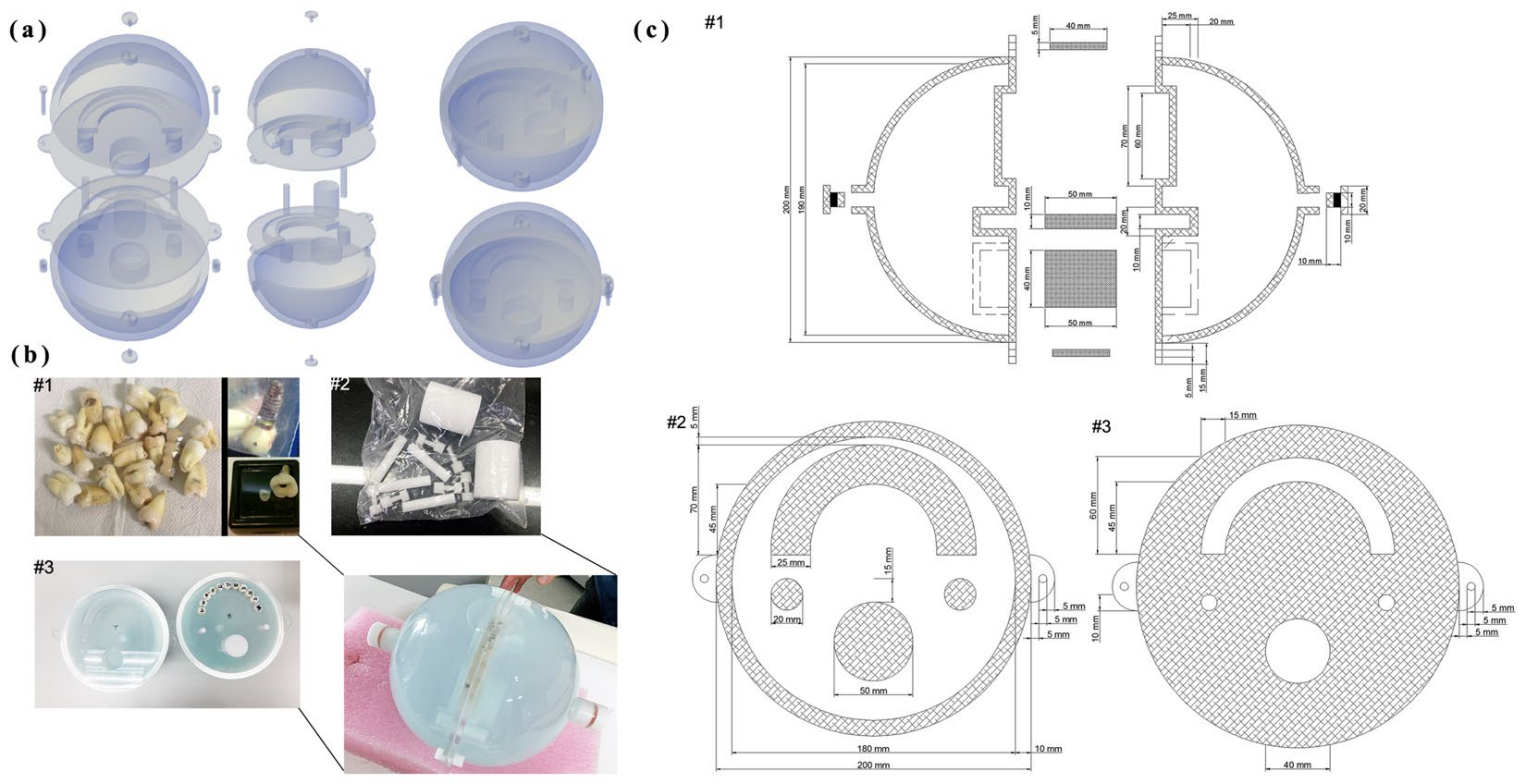
Phantom design and appearance. (**a**) The dedicated MR/CT oral phantom model and geometry with a functional decomposition. (**b**. #1 and **b**. #2) The circular phantom which is shaped with the anatomical structures of a human head and neck. (**b**. #3) Teeth are inserted into the cylindroid thermoplastic, and are interchangeable with bone-like Teflon. (**c**. #1) Longitudinal and planar sectional view of (**c**. #2) inner and (**c**. #3) outer position.

As all other imaging phantoms, the developed CT/MR oral phantom was designed to test the limitation of imaging systems and were compatible with multimodality imaging. It was clearly impractical and dangerous to place a human being in the beam path to take measurements. The physical design of the developed phantom was dictated by the need to accurately simulate the density of various human tissues for CT scanning. Two hemispherical containers (outer diameter, 200 mm; inner diameter, 180 mm; thickness, 10 mm) were made using acrylic material (density, 1.20 g/cm^3^) that was resistant to magnetic field (Fig. 5).

To prevent water from leaking, and air from entering the construction, a water-tight lid (outer diameter, 20 mm; inner diameter, 10 mm) was used for the upper and lower hemispherical containers, consisting of an insoluble plug (polylactic acid; height, 10 mm; diameter, 10 mm) with low susceptibility artifacts. The respective inner portions were formed to allow the insertion of bone-like Teflon^®^ into the inner part of the phantom. In order to mimic the human head and neck, columnar Teflon was inserted into each of the three 10-mm and 40-mm holes, to represent spine and mandible. The phantom consisted of CT materials including Teflon, which can be of up to 890 HU, with a density value of 2.16 g/cm^3^, mimicking bony structures, such as cervical vertebrae. The teeth were inserted into the cylindroid polymorph plastic teeth retainer, and each tooth was freely interchangeable. Polymorph plastic was used for the manufacture of each cylindroid tooth and dental restoration, at a temperature of 65 °C. For more accurate mimicking, the developed phantom included the teeth material from removed real human wisdom teeth (the teeth were removed as a part of routine clinical practice), and implants made with products that are commercially used in the dental clinic, such as pure titanium and titanium alloys. Both hemispherical containers were filled with copper sulfate (CuSO_4_, 0.7 g/L) diluted with water to reduce the T1 relaxation time. Reducing the T1 relaxation time allows stronger signal acquisition, and decreases temperature dependence^15^. Sodium chloride (NaCl, 3.6 g/L) was selected to ensure conductivity similar to that of the human body^30^. In this study, 11 teeth were set in the prepared teeth retainer. The first set consisted of the phantom containing 10 normal teeth, and 1 dental restoration was inserted in the left side of the teeth retainer. The second set consisted of the phantom containing 9 normal teeth, and 2 dental restorations inserted in the top middle portion of the teeth retainer, to evaluate the geometric accuracy.

### Acquisition of CT/MR images

All MR measurements were made using a human 3.0 T MR scanner (Achieva Tx 3.0 T, Philips Medical Systems, Netherlands), with a 32-channel sensitivity encoding head coil. The phantom was aligned with the geometric center of the two hemispherical containers. Appropriate coil setup was important for reducing artifacts. To simulate the dental position, the test object was placed in the containers that were perpendicular to the static magnetic field. The normal dental images were used as a reference. After imaging at the three orthogonal planes, SE with multi-bandwidths and GRE sequences with multiple TEs were conducted. The MR scanning parameters were as follows: (1) SE sequence: repetition time (TR), 500 ms; TE, 20 ms; bandwidths, 145, 160, 175, 190, 240, 290, 340, 390, 440, and 490 Hz/px; flip angle (FA), 90°; slice thickness, 1 mm; matrix, 560 × 560; FOV, 240 mm; gap, 1 mm; number of signal averages (NSA), 16; (2) GRE sequence: TR, 500; multiple TEs, 8, 10, 15, 20, 25, 30, 35, 40, 45, and 50 ms; bandwidth, 130 Hz/px; FA, 30°; slice thickness, 1 mm; matrix, 512 × 512; FOV, 240 mm; gap, 1 mm; and NSA, 16. In all sequences, the phantom was imaged with dental restorations (left and top of the dental arch) and without them (controls). Analysis of the images made using MATLAB Image Processing Toolbox (MATLAB, The MathWorks, Inc., Natick, MA, USA). Multiple sets of Digital Imaging and Communications in Medicine (DICOM) data were acquired by applying the standard image processing function, and calculated using pixel values of the area imaged. For the CT images, high-resolution images were acquired using a 16-slice CT scanner (Mx8000 16 IDT; Philips Medical Systems, Andover, MA, USA) with 120 kVp, 190 mA, slice thickness of 1 mm, and FOV of 240 mm, similar to the MR imaging parameters.

### Segmentation and registration

**Figure 6:**
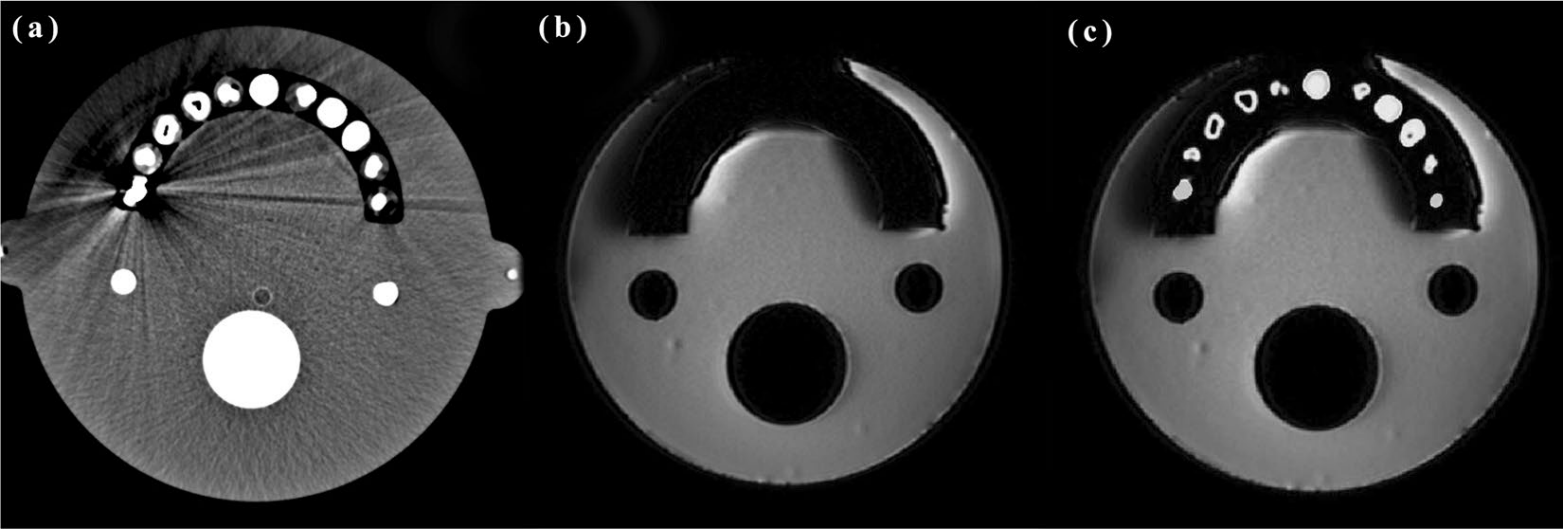
Image slices from scans of the CT/MR oral phantom with left teeth restoration. (**a**) CT, (**b**) MR (GRE sequence: TR, 500; TE, 50 ms; bandwidth, 130 Hz/px), (**c**) and image after CT/MR registration.

The first step was to segment the metal and normal tooth regions using a suitable threshold, from an initial CT image reconstructed without metal artifact-free. The second step was to acquire the regions of metal from the MR images. Subsequently, the CT images were applied to the MR images, which are shown in Fig. 6. Automatic threshold segmentation was used to segment the regions of interest from the CT images. Region competition was based on the threshold of the image intensities. The intensity values of CT images may change or remain unchanged, depending on whether the intensity was within the threshold range (1200–3500 HU) that covers the oral region. Based on these specifications, a range of intensities was designated as the foreground or object intensities. The intensities outside this range were defined as background. All image data were exported in DICOM format to a stand-alone windows workstation. The MR images were down-sampled to a 512 × 512 matrix size from the original 560 × 560 matrix size. This down-sampling was necessary since the automatic registration algorithm was a multi-resolution algorithm that required the initial image dimensions to be aligned.

**Figure 7:**
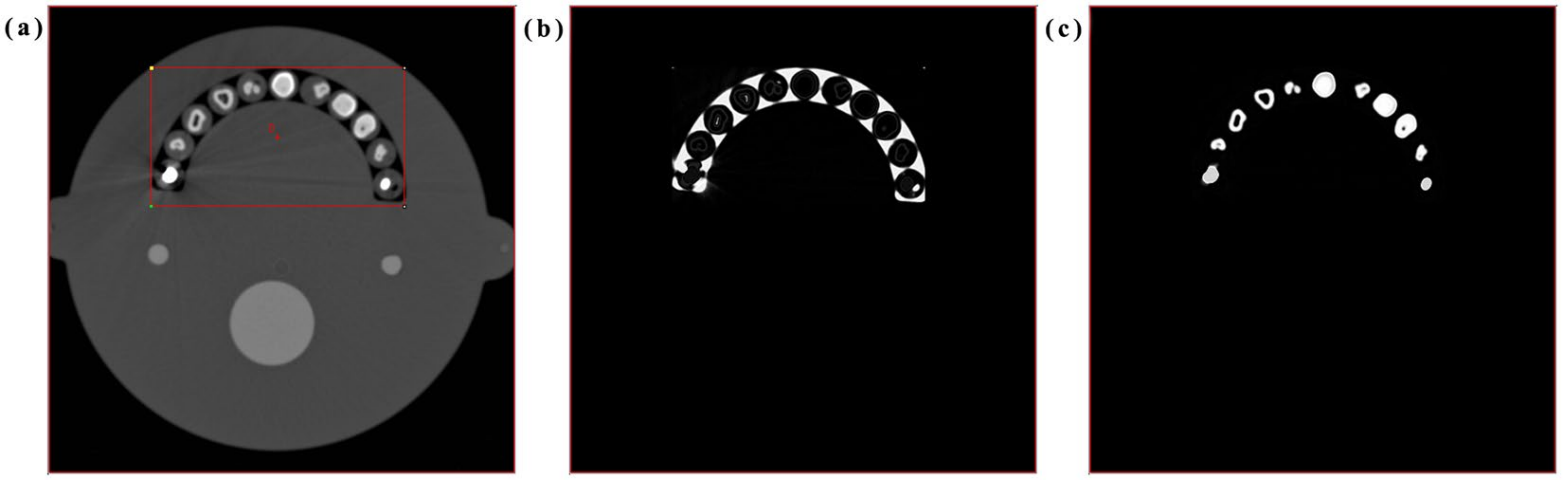
Results of brain phantom image with left teeth restoration that segmented based on FCM algorithm (**a**) select the interest region (teeth including restorations) from original CT slice, (**b**) FCM, and (**c**) FCM with thresholding.

The FCM algorithm is one of the most commonly used image segmentation methods, allowing more information to be retained from the original image than the crisp/hard segmentation methods. The FCM clustering algorithm was first introduced and developed by Dunn^31^ and Bezdek^32^. The algorithm is an iterative clustering method that produces an optimal c partition by minimizing the weighted within-group sum of squared error objective function J_F_ (1):

1 $${J}_{F}=\sum _{a=1}^{n}\sum _{b=1}^{c}\,{u}_{ab}^{F}{\Vert {x}_{a}-{c}_{b}\Vert }^{2},\,1\le {\rm{F}} < \infty $$

where F is any real number greater than 1, u_ab_ is the degree of membership of x_a_ in the cluster b, x_a_ is the a^th^ of d-dimensional measured data, c_b_ is the d-dimension center of the cluster, and $\Vert \ast \Vert$ is any norm expressing the similarity between any measured data and the center. This iteration will stop when max _ab_ $\{|{u}_{ab}^{(q+1)}-{u}_{ab}^{(q)}|\} < \varepsilon$, where $\varepsilon$ is the termination criterion between 0 and 1, whereas *q* are the iteration steps^33,34^. The detailed process is shown in Fig. 7. The registered images were used to analyze the image quality and quantify the loss of information during the distortion process^35,36^. Mathematically defined measures used include the widely used MSE, RMSE, PSNR, cross-correlation, MAE, and SSIM characteristics. The MSE and the PSNR were used to evaluate the quality of a reconstructed image. The MSE represents the cumulative squared error between the compressed and the original image, whereas PSNR represents the peak error. The RMSE is the standard deviation of the prediction errors which are a measure of how far from the regression line the data points are. The statistic MAE is the average of all absolute errors, and it is less sensitive to the magnitude error because it does not square the errors. Lastly, SSIM is a method for measuring the similarity between two images, and it has greater accuracy and consistency than MSE and PSNR. If the value of SSIM is close to 1, two images are nearly identical. On the contrary, if the value of SSIM is close to 0, the correlation between two images is low.

2 $${\rm{SSIM}}({\rm{x}},{\rm{y}})=\frac{(2{\mu }_{x}{\mu }_{y}+{c}_{1})(2{\sigma }_{xy}+{c}_{2})}{(2{\mu }_{x}^{2}+{\mu }_{y}^{2}+{c}_{1})({\sigma }_{x}^{2}+{\sigma }_{y}^{2}+{c}_{2})}$$
